# A voting mechanism-based linear epitope prediction system for the host-specific *Iridoviridae* family

**DOI:** 10.1186/s12859-019-2736-2

**Published:** 2019-05-01

**Authors:** Tao-Chuan Shih, Li-Ping Ho, Jen-Leih Wu, Hsin-Yiu Chou, Tun-Wen Pai

**Affiliations:** 1 Department of Computer Science and Engineering, National Taiwan Ocean University, Keelung, Taiwan; 2 Center of Excellence for the Oceans, National Taiwan Ocean University, Keelung, Taiwan; 3 Department of Bioscience and Biotechnology, National Taiwan Ocean University, Keelung, Taiwan; 4 Institute of Cellular and Organismic Biology, Academia Sinica, Taipei, Taiwan; 5 Department of Aquaculture, College of Life Science, National Taiwan Ocean University, Keelung, Taiwan; 6 Department of Computer Science and Information Engineering, National Taipei University of Technology, Taipei, Taiwan

**Keywords:** *Iridoviridae*, Linear epitope, Propensity scales, Host specificity, Group feature

## Abstract

**Background:**

The *Iridoviridae* family is categorized into five genera and clustered into two subfamilies: *Alphairidovirinae* includes *Lymphocystivirus*, *Ranavirus* (GIV), and *Megalocystivirus* (TGIV), which infect vertebrate hosts and *Betairidovirinae* includes *Iridovirus* and *Chloriridovirus,* which infect invertebrate hosts. Clustered *Iridoviridae* subfamilies possess host-specific characteristics, which can be considered as exclusive features for *in-silico* prediction of effective epitopes for vaccine development. A voting mechanism-based linear epitope (LE) prediction system was applied to identify and endorse LE candidates with a minimum length requirement for each clustered subfamily

**Results:**

The experimental results showed that four conserved epitopes among the *Iridovirideae* family, one exclusive epitope for invertebrate subfamily and two exclusive epitopes for vertebrate family were predicted. These predicted LE candidates were further validated by ELISA assays for evaluating the strength of antigenicity and cross antigenicity. The conserved LEs for *Iridoviridae* family reflected high antigenicity responses for the two subfamilies, while exclusive LEs reflected high antigenicity responses only for the host-specific subfamily

**Conclusions:**

Host-specific characteristics are important features and constraints for effective epitope prediction. Our proposed voting mechanism based system provides a novel approach for in silico LE prediction prior to vaccine development, and it is especially powerful for analyzing antigen sequences with exclusive features between two clustered groups.

## Background

Members of the family *Iridoviridae* are large icosahedral viruses that contain a single molecule of double-stranded DNA with genome sizes ranging from 104 to 220 Kbp [1]. The virus structure contains three domains, including an outer proteinaceous capsid, an intermediate lipid membrane, and a central core. Some viruses possess an outer envelope, and the outer capsid is composed of major capsid protein (MCP), which appears to be highly conserved among the family and possesses surface binding sites interacting with the surfaces of host’s cells [2]. The family *Iridoviridae* is categorized into five genera and clustered into two subfamilies (Table 1). The first *Betairidovirinae* subfamily, also referred as invertebrate iridescent viruses, contains both *Iridovirus* and *Chloriridovirus* genera, which infects invertebrate hosts such as insects and crustaceans. The second subfamily of *Alphairidovirinae,* also called vertebrate iridoviruses, includes *Megalocystivirus* [3] and *Lymphocystivirus* [3, 4] which infects fish only and *Ranavirus* [3] genus that infects fish, amphibian, and reptiles [5, 6]. In fact, vertebrate iridoviruses have caused high mortality of farmed fish and have led to huge economical lost [7]. For example, grouper is an important cultured species, which has suffered from vertebrate iridovirus infection in recent years. The mortality of infected groupers was up to 60% for length 2–4 in. [8]. Due to high-density farming, the virus can be horizontally transferred once one fish is infected by vertebrate iridoviruses to cause severe damage. Surviving fish might retain the pathogens and continuously spread the virus to other health fish within the same pool [8, 9]. The prevention of vertebrate iridovirus infection has become an important task in fish farming. However, despite several injectable and oral commercial vaccines, the prevention results of specific vaccines have not been satisfactory for high-density farming. Therefore, a more effective immunization strategy and comprehensive vaccine development for different vertebrate iridoviruses have become important for challenging agriculture environments.

In general, immunobiologists have developed an integrated method for vaccine development based on analyzing protein sequences and structures of target viruses [10]. However, only few protein structures have been currently resolved for the *Iridoviridae* family, none of which are associated with the outer capsid proteins. Therefore, the prediction of MCP structures will be performed for surface analysis to facilitate the verification of the predicted LEs. B-cells play an important role in the immune system, and receptors on the cell membrane allow B-cells to bind to specific antigens [11]. Antigen proteins are usually too large to bind as a whole to any receptor. Hence, partial antigen segments located on surface areas called epitopes are bound by specific antibodies. Epitopes are categorized into two different types: linear epitope (LE) and conformational epitope (CE). An LE is a continuous sequence of amino acids contacting the receptors directly. By contrast, a CE is composed of discontinuous primary peptides, which are close neighbors on the three-dimensional surface. Generally speaking, more antibodies recognize CEs than LEs [12]. This is mainly due to native conformations being maintained to retain protein function [13]. However, the CE prediction requires antigenic structures to be resolved in advance for conformational region analysis. Due to this requirement, the LE prediction is a popular approach when the corresponding antigenic structures do not exist. In order to predict effective LEs as vaccine candidates, the predicted peptides should effectively elicit antibodies from specific hosts that recognize the antigens and provide protection against infection [14]. In addition, the selected peptides for vaccine design should be ideally conserved across different stages of the pathogen and possess binding affinity for the major populations of specific hosts [13]. In this study, we developed a voting mechanism-based computational system for linear epitope prediction, using the family *Iridoviridae* as an example for biological validation. The proposed system selected five existing epitope prediction tools, including LBTOPE [15], BepiPred [16], BCPREDS [17], ABCPred [18], and LEPS [19]. Corresponding predicted epitopes for each tool were obtained, aligned, and selected through a voting procedure. The LBTOPE retrieved experimentally validated B-cell epitopes as well as non B-cell epitopes from the Immune Epitope Database (IEDB), and the system discriminated all candidate segments using an SVM classifier on various features, such as binary profile, dipeptide composition, and amino acid pair (AAP). Jespersen and researchers developed the BepiPred2.0 based on the random forest algorithm, and the system trained the epitope dataset annotated from antibody-antigen protein structures. BepiPred predicted epitopes derived from solved 3D structures and a large collection of linear epitopes downloaded from the IEDB database. EL-Manzalawy and researchers proposed the BCPREDS, which is based on SVM approaches, and utilized five different kernel methods and five-fold cross-validation of homology-reduce datasets that contained 701 linear B-cell epitopes and non-epitopes. The predicted epitopes were verified by predicted structures and structural alignment. In terms of the artificial neural network approach, ABCPred utilized a recurrent neural network (RNN) mode to train and test using different input window lengths and hidden units. The final network yielded an overall prediction accuracy of 65.93% through a five-fold cross-validation. The last LEPS prediction system was based on physicochemical propensity feature analysis and SVM classification. Peptides with globally or locally high physicochemical propensities were first identified as primitive linear epitope candidates. Subsequently, candidates were further validated through SVM classifiers according to collected known epitopes. After the target antigenic sequences were individually evaluated by these five LE predictors, each residue or a continuous segment within the target antigenic sequences was predicted as epitope or non-epitope residues. Therefore, a multiple sequence alignment should be performed to discover the conserved or exclusive LE candidates according to the clustered group features. A voting rule approach that incorporates surface structure evaluation is proposed in this study to reconfirm identified conserved and/or exclusive LE candidates. All designed prediction procedures and experimental materials are introduced and discussed in the following sections.

## Materials and methods

### Data collection

Five genera have been phylogenetically classified in the *Iridoviridae* family based on biological properties of the viruses, and representative species from each genus were selected according to the previously published results [5]. Uncertain taxonomic statuses of tentative species to the five established iridovirid genera were deliberately not considered in this study. Both *Lymphocystivirus* and *Chloriridovirus* genera contain one recognized virus species, LCDV-1 and IIV-3, and these two viral species were solely selected as the representative sequences. The *Iridovirus* genus is consisted of two clearly classified species including IIV-6 and IIV-1 species. However, there is no MCP sequence could be found for IIV-1, so the IIV-6 species was selected as the representative species for *Iridovirus* genus. As for the *Megalocystivirus* and *Ranavirus* genera, each genus contains several recognized species by ICTV Subcommittee (International Committee on Taxonomy of Viruses). Since TGIV (*Megalocystivirus*) and GIV (*Ranavirus*) were host-specific antigens for groupers, these two virus species were selected as representative antigen sequences for the following biological experiment validation. All corresponding major capsid protein sequences were retrieved from NCBI [20] and Uniport [21]. To observe their corresponding virtual structures for surface verification, we applied Phyre2 [22] to predict the three-dimensional model for each capsid protein. The genus,representative species, and specific infected hosts are shown in 1.

### System flowchart

**Fig. 1:**
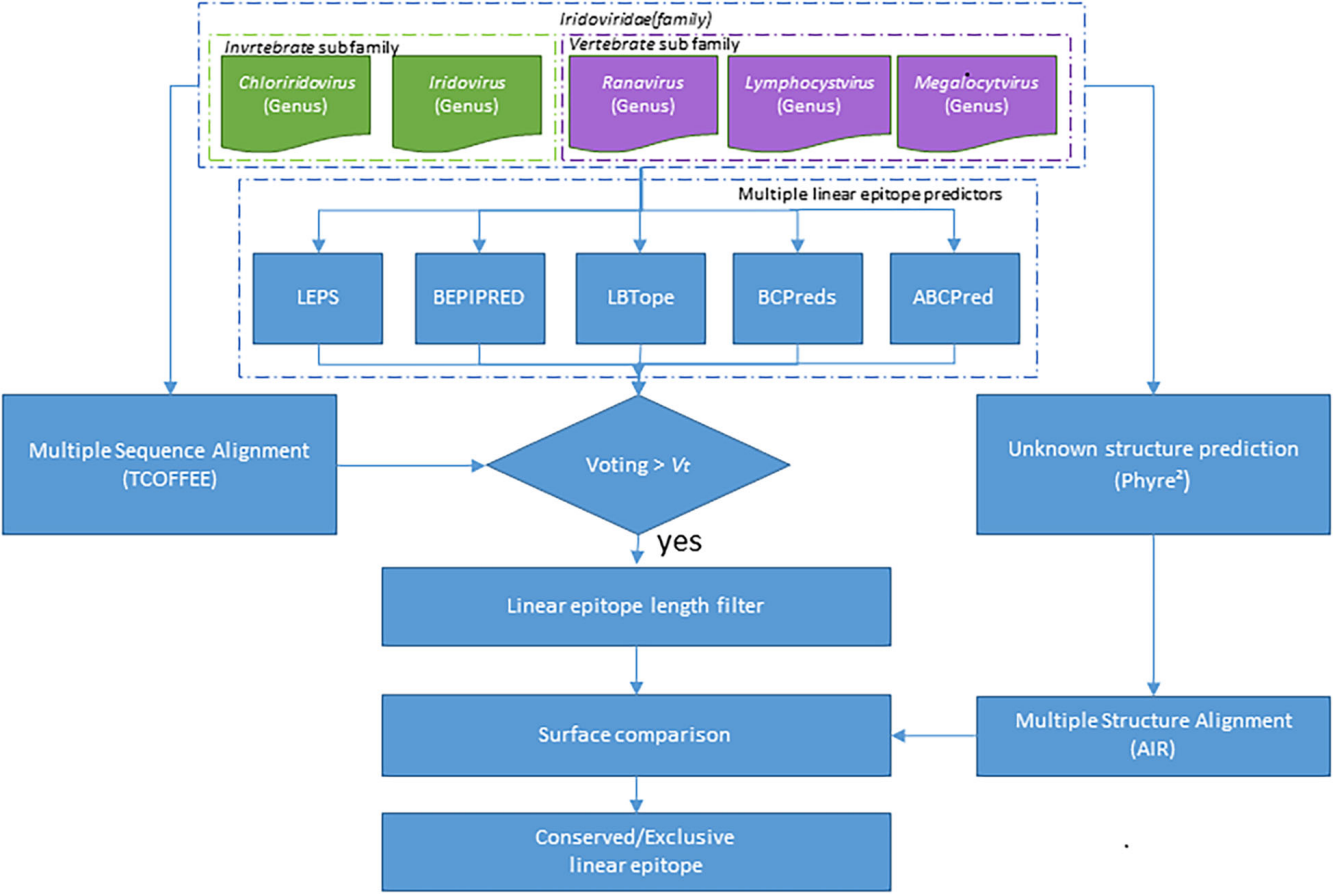
System flow chart

The designed voting mechanism-based LE prediction system is shown in Fig. 1. Representative MCP sequences from five different representative genera in the *Iridoviridae* family were input as query data. The system delivered five representative MCP sequences to T-Coffee [23] for performing multiple sequence alignment, and the Phyre2 was applied for structure prediction of each sequence. As the host specificity of each species in the *Iridoviridae* family were previously known, two groups, including vertebrate iridovirus subfamily (*Megalocystivirus*, *Ranavirus*, and *Lymphocystivirus*) and invertebrate iridovirus subfamily (*Chloriridovirus* and *Iridovirus*), were clustered according to host specificity. Five existing epitope prediction tools, including LBTOPE, BepiPred, BCPREDS, ABCPred, and LEPS, were utilized to predict LEs for each representative antigenic sequence. As a result, several conserved and/or exclusive structural segments between these two clustered subfamilies were determined, of which unaligned protein sequences and conformational variances may lead to host specificity. Nevertheless, the conserved epitope segments within the same clustered antigen group may play important roles for antibody binding with the same specific host species groups. To present multi-function of the proposed system, the five genera were applied as two different trials for LE prediction, including (1) the whole *Iridoviridae* family as a single group for conserved LEs and (2) two clustered groups (vertebrate and invertebrate iridovirus subfamilies) for detecting conserved and exclusive LEs. Each antigenic sequence in the grouped clusters was analyzed using the five linear epitope prediction tools. These published web tools have been designed to identify each antigenic residue as an epitope or non-epitope residue. The results were encoded by a binary format, one for prediction as an epitope candidate residue and zero for a non-epitope residue. Hence, each residue in an antigen sequence possessed a total score from the five different prediction tools. According to various feature aspects of the different prediction tools, an accumulated higher score represented a higher confidence to suggest the residue as an epitope residue. To evaluate the conserved and/or exclusive characteristics among the whole *Iridoviridae* family, the final epitope scores were accumulated based on aligned residue positions from multiple sequence alignments. Though the sequence residue contents of an aligned position were different for distinct subfamilies due to evolution, the physicochemical and geometrical characteristics of aligned positions were mostly preserved. Hence, regarding the multiple sequence aligned results, if the accumulated epitope score of a residue from all prediction tools was higher than one half of the maximum score from all predicted residues, then the residue was considered as a predicted epitope candidate. The next step was to concatenate the continuous individual epitope candidates in neighboring relationships for an epitope with a minimum length requirement. Finally, both conserved and exclusive LEs for both vertebrate iridovirus and invertebrate iridovirus subfamilies were carefully enumerated and reconfirmed.

**Table 1:** Selected genera of the *Iridoviridae* family and infected host species

Genus	Selected species	Hosts
*Lymphocystivirus*	*Lymphocystis* disease virus 1 (LCDV-1)	Fish species (vertebrate)
*Megalocystivirus*	Taiwan grouper *Iridovirus* (TGIV)	Fish species (vertebrate)
*Ranavirus*	Grouper *Iridovirus* (GIV)	Amphibians, reptiles, and fish species (vertebrate)
*Iridovirus*	Invertebrate iridescent virus-6 (IIV-6)	Insects, crustaceans (invertebrate)
*Chloriridovirus*	Invertebrate iridescent virus-3 (IIV-3)	Mosquitoes (invertebrate)

## Results

### LE prediction

**Fig. 2:**
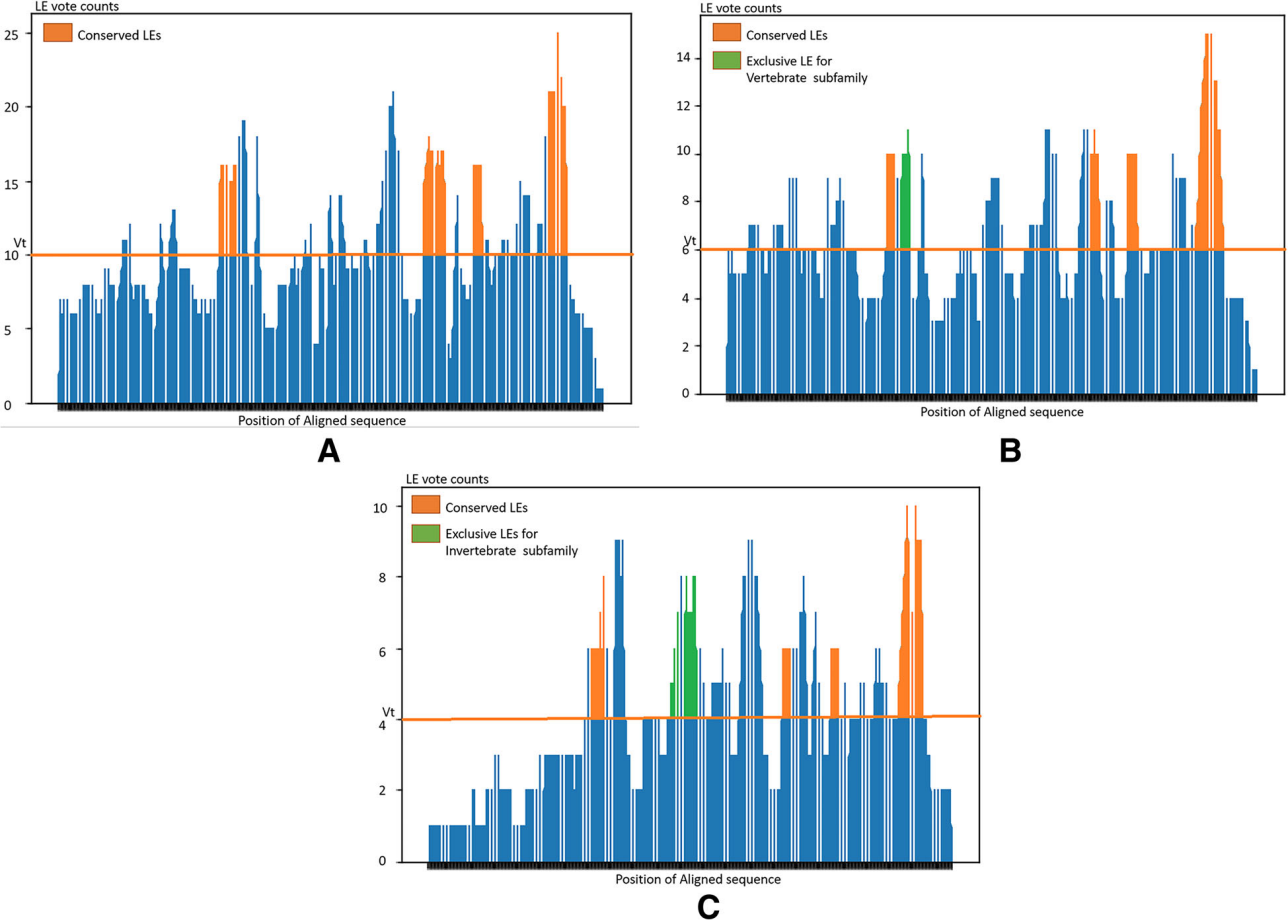
Locations of predicted LEs and corresponding residue contents. Residues possessing voting counts greater than the threshold setting are considered as candidate epitope residues and shown in orange for conserved epitopes and green/purple for unique epitopes. X-axis represents the position of residues according to multiple sequence-aligned results. Y-axis is the total voting counts of residues estimated by the five different LE predictors. (**a**) Voting results for all *Iridoviridae* families (total length: 479, V*t*: 10). Four predicted LEs with lengths greater than 7 residues are shown in orange, and the residue contents are listed in the table. (**b**) Voting results for the VIV subfamily only (total length: 472, V*t*: 6). Five predicted LEs with lengths greater than 7 residues are shown and listed in the table, including four conserved LEs in orange and one exclusive LE in green. (**c**) Voting results for the IIV subfamily only (total length: 468, V*t*: 4). Six predicted LEs with lengths greater than 7 residues are shown and listed in the table, including four conserved LEs in orange and two exclusive LEs in green

The results of LE prediction were illustrated by two different trials: (1) import all five *Iridoviridae* family members as a single group to identify conserved epitopes and (2) import two host-specific groups of invertebrate iridovirus (IIV) subfamily and vertebrate iridovirus (VIV) subfamily to discover conserved and exclusive linear epitopes. Based on the designed system, all target pathogen sequences could be imported and assigned as a single or two groups for the antigenic feature analysis. A consensus voting system for LE prediction based on five linear epitope tools was performed on the target sequences individually. To make the final decision, a default threshold setting of half of the maximum voting counts for an epitope residue candidate was applied according to multiple sequence alignment results. In addition, continuous peptide segment with lengths longer than 7 consecutive epitope candidate residues were considered as the final predicted LEs. The voting count distributions of three trials for the aligned sequences are shown in Fig. 2, and the dynamic threshold settings are depicted with orange lines. The final predicted LEs with lengths shorter than 7 or longer than 25 amino acids were discarded, as B cell epitopes are rarely outside of these ranges [24]. All candidates of conserved and/or exclusive LEs for the *Iridoviridae* family, VIV subfamily, and IIV subfamily are shown in Table 2 and Table 3 respectively.

**Table 2:** Predicted conserved LEs and corresponding RMSDs of virtual structures by taking the whole *Iridoviridae* family as a single group

Iridoviridae groups	Predictive LEs of representative peptide	Residue Location	RMSDs of structural alignment (Å)
*Iridoviridae* family (Conserved LEs)	KAVGYDNMIGN	*Iridovirus*:145~155	0.533
	KRNGYDNMIGN	*Chloriridovirus*:46~56	
	KRIGYDNMIGN	*Lymphocystivirus*:146~156	
	KRIGYDNMIGN	*Megalocystivirus*:146~156	
	KQSGYNKMIGM	*Ranavirus*:141~151	
	SNYTTASPVITSTS	*Iridovirus*:323~336	1.313
	SNYGTSSPVVSGTS	*Chloriridovirus*:222~233	
	SNYTSSSPVIFDGG	*Lymphocystivirus*:315~327	
	SNYTCVTPVNGPGN	*Megalocystivirus*:320~332	
	SNYTAASPVYVNNK	*Ranavirus*:311~323	
	RLNHMGSD	*Iridovirus*:360~367	0.9
	RLGTMGSD	*Chloriridovirus*:258~265	
	RLNEMGSE	*Lymphocystivirus*:352~359	
	RLANMGVE	*Megalocystivirus*:356~363	
	RLHQMGVD	*Ranavirus*:346~353	
	GAAGTGPAGSGQNFPQT	*Iridovirus*:424~442	1.122
	ASTGAGDGAGANYNQS	*Chloriridovirus*:324~339	
	TAGGNGGNTSGYKDAQK	*Lymphocystivirus*:415~434	
	AAAGGGNNNSGYNEPQR	*Megalocystivirus*:419~438	
	TAAGGGGNGTGYTVAQK	*Ranavirus*:409~428

**Table 3:** Predicted conserved and exclusive LEs for two clustered groups: vertebrate and invertebrate iridovirus subfamiliese. (*N/A represents the predicted segments possessing missing residues among the five virtual structures)

*Iridoviridae* groups	Predictive LEs of representative peptide		Residue Location	RMSDs of structural alignment(Å)
*Iridoviridae* family (Conserved LEs)	KAVGYDNMIGN	(IIV)	*Iridovirus*:145~155	1.233
	KRNGYDNMIGN		*Chloriridovirus*:46~56	
	KRIGYDNMIGN	(VIV)	*Lymphocystivirus*:146~156	0.063
	KRIGYDNMIGN		*Megalocystivirus*:146~156	
	KQSGYNKMIGM		*Ranavirus*:141~151	
	SNYTTASPVITSTT	(IIV)	*Iridovirus*:323~336	2.685
	SNYGTSSPVVSGTS		*Chloriridovirus*:222~233	
	SNYTSSSPVIFDGG	(VIV)	*Lymphocystivirus*:315~327	0.268
	SNYTCVTPVNGPGN		*Megalocystivirus*:320~332	
	SNYTAASPVYVNNK		*Ranavirus*:311~323	
	RLNHMGSD	(IIV)	*Iridovirus*:360~367	1.792
	RLGTMGSD		*Chloriridovirus*:258~265	
	RLNEMGSE	(VIV)	*Lymphocystivirus*:354~359	0.231
	RLANMGVE		*Megalocystivirus*:356~363	
	RLHQMGVD		*Ranavirus*:346~353	
	GAAGTGPAGSGQNFPQT	(IIV)	*Iridovirus*:424~442	2.385
	ASTGAGDGAGANYNQS		*Chloriridovirus*:324~339	
	TAGGNGGNTSG	(VIV)	*Lymphocystivirus*:418~428	0.308
	AAAGGGNNNSG		*Megalocystivirus*: 422~432	
	TAAGGGGNGTG		*Ranavirus*:412~422	
VIV (Exclusive LEs)	TIDMTQPVDS TSDMTNPTPA RSDLVGGITN		*Lymphocystivirus*:157~166	N/A*
			*Megalocystivirus*: 157~166	
			*Ranavirus*:152~161	
IIV (Exclusive LEs)	VALPTAALPYNE VSLPTAALPYNE		*Iridovirus*:193~203	2.683
			*Chloriridovirus*:92~102	
	VASQTVVPVVG TTGNPYQTIDV		*Iridovirus*:226~236	N/A*
			*Chloriridovirus*:125~135	

For the first trial of five *Iridoviridae* family members as a single group, there were four predicted LEs satisfying the criteria. Predicted peptides for each iridovirus sequence and corresponding aligned residue positions are listed in Fig. 2(a) and Table 2. In the second trial clustering five *Iridoviridae* family members into two groups, four conserved LEs were predicted as the results of the first trial (identical conserved LEs in both Table 2 and Table 3), one exclusive LE were predicted for the VIV subfamily (Fig. 2(b) and Table 3), and two exclusive LEs were predicted for the IIV subfamily (Fig. 2(c) and Table 3). All these detected LEs possessed high antigenicity characteristics, which were supported by five linear epitope predictors.

### Conserved and diverse structural segments of predicted LEs

**Fig. 3:**
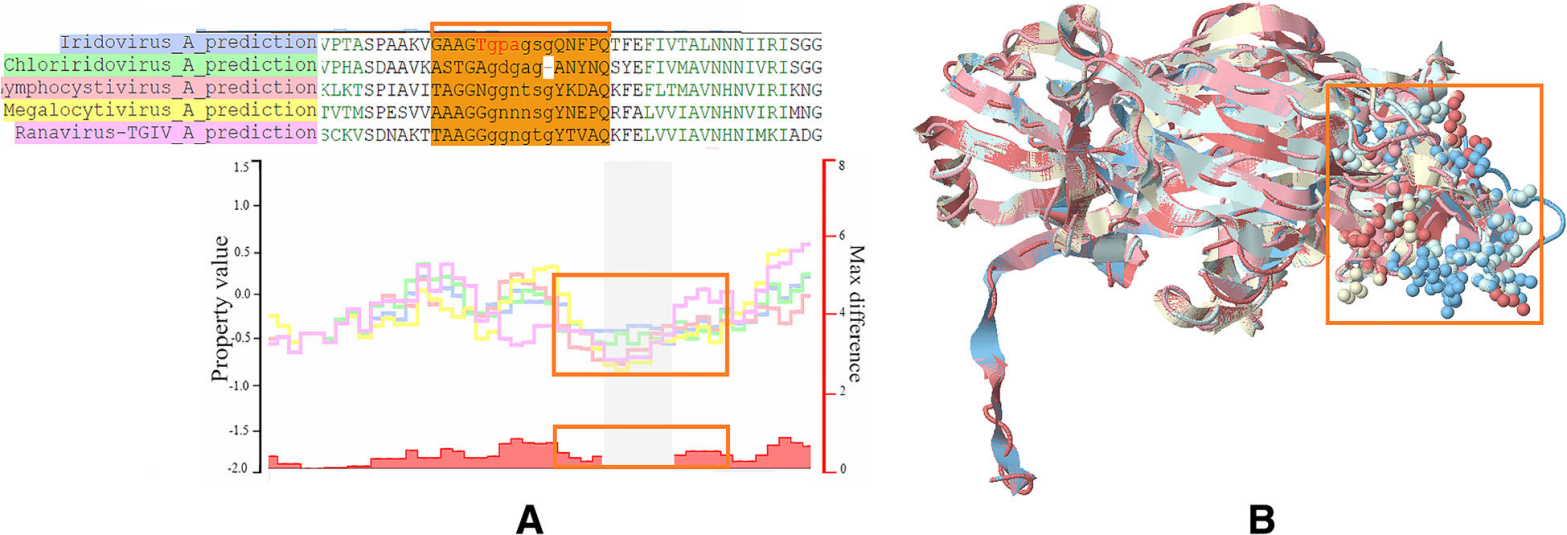
Predicted LEs for both VIV and IIV iridoviruses. Five predicted representative structures were multiple aligned for observing structural variations: (**a**) aligned sequence segments and corresponding RMSD variations; (**b**) structural representation of five aligned *Iridoviridae* MCPs and selected segments in atomic representation

To explore the structural variations of predicted LEs among different iridoviruses, the structure alignment of detected epitope candidates was performed by the Alignment Incorporate Refinement (AIR) system (unpublished system). AIR can measure the special similarity and molecular evolutionary relationship among protein structures by calculating core residue-based root-mean-square-deviation (RMSD) variations in atomic distances. In addition, the physicochemical variations could be easily viewed through the aligned structural conformation. The aligned results of the designed clustered experiments and the corresponding measurements by AIR are shown in both Table 2 and Table 3, and an example of structure aligned result is shown in Fig. 3 with visualized RMSD variations. RMSD variation was used to measure the geometrical similarity of aligned three-dimensional structures regarding the spatial distance of corresponding residues. In this study, RMSD measurements were applied for searching conserved and diverse structural segments between invertebrate *Iridoviridae* and vertebrate *Iridoviridae* subfamilies. The higher RMSDs represented lower structural similarity of the predicted LE segments. We assessed one of the predicted epitopes of “TAGGNGGNTSGYKDAQK” (from *Lymphocycstivirus*) as an example since the segments possessed a relative higher epitope score. The segment was predicted as a conserved linear epitope for the *Iridoviridae* family. Five aligned predicted LE segments possess an average RMSD of 1.122 (Å), and these LE segments can be selected and viewd through a 3D view page which utilizes Jmol open-sources (a Java viewer for 3D chemical structures). Multiple predicted LE segment alignment and their corresponding structural aligned results from predicted protein structures can be viewed and compared through the AIR system simultaneously. Surface properties of predicted LEs and structural diversity among various viruses could be evaluated and confirmed for the next step of vaccine design and development.

### ELISA assays for host-specific antigenicity and cross antigenicity tests

To validate immune responses evoked by the predicted LEs from both VIV and IIV subfamilies, ELISA assays were performed to evaluate the strength of antigenicity and cross antigenicity. According to the predicted results shown in Table 3, we selected exclusive LEs of TIDMTQPVDS for *Lymphocystivirus*_157–166_, TSDMTNPTPA for *Megalocystivirus*_157–166_, and RSDLVGGITN for *Ranavirus*_152–161_ from the VIV subfamily for antigenicity tests, and for the IIV subfamily, we selected the four predicted exclusive LEs of VALPTAALPYNE for *Iridovirus*_193~203_, VSLPTAALPYNE for *Chloriridovirus*_92~102_, VASQTVVPVVG for *Iridovirus*_226–236_, and TTGNPYQTIDV for *Chloriridovirus*_125–135_ for cross antigenicity tests. All selected LE segments were synthesized by GeneScript Company (USA) for biological validation. After synthesis procedures, the synthesized epitopes were used as antigen in the ELISA assay respectively. Rabbit anti-rTGIVmcp and anti-rGIVmcp antibodies were individually prepared by the local bio-supplier. The secondary antibody Goat anti-rabbit IgG(h + l) conjugated with horseradish peroxidase (HRP) was purchased from Bethyl Laboratories (TX, USA). Briefly, 10 μg of synthesized epitopes were coated on a 96-well microplate with coating buffer (pH 9.6, Sigma-Aldrich, USA). All procedures were performed in accordance to the manufacturer’s protocol [25]. After hybridization, substrate TMB (3,3′,5,5′-tetramethylbenzidine, Amersco, USA) was added for HRP detection and read at 630 nm on an ELISA reader. The ELISA results were analyzed by ANOVA (Analysis of Variance) in the GraphPad Prism 5.0 biological statistical software (GraphPad Software, Inc.).

**Fig. 4:**
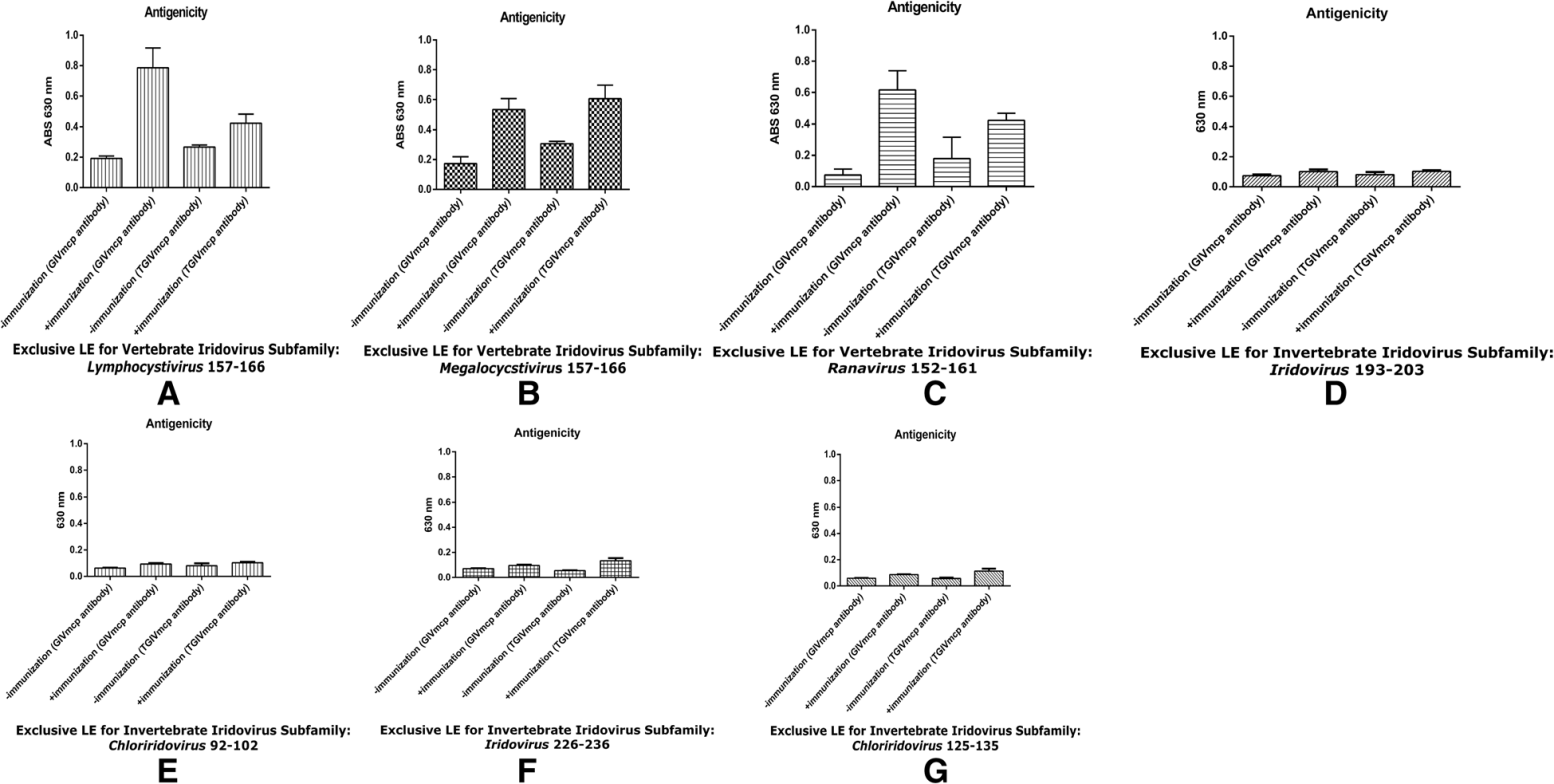
Antigenicity test by ELISA assays. 10 μg of different synthetic peptides were coated on a 96-well microplate, respectively. For antigenicity and cross antigenicity tests, all synthetic peptides were hybridized by rabbit anti-rTGIVmcp or rabbit anti-rGIVmcp antibodies and detected by goat anti-rabbit IgG (h + l) conjugated horseradish peroxidase (HRP) antibody. The figures of **a**, **b**, and **c** represent the responses of synthetic peptides for VIV subfamily including *Lymphocystivirus*_157–166_, *Megalocystivirus*_157–166_, and *Ranavirus*_152–161_ (exclusive LEs for VIV group), respectively; the figures of **d**, **e**, **f**, and **g** represent the responses of synthetic peptides for IIV subfamily including *Iridovirus*_193–203_, *Chloriridovirus*_92–102,_ *Iridovirus*_226–236,_ and *Chloriridovirus*_125–135_ (exclusive LEs for IIV group), respectively. **-immunization (GIVmcp antibody): before immunization by rGIVmcp (pre-immune serum). +immunization (GIVmcp antibody): after immunization by rGIVmcp (immunized serum). -immunization (TGIVmcp antibody): before immunization by rTGIVmcp (pre-immune serum). +immunization (TGIVmcp antibody): after immunization by rTGIVmcp (immunized serum). ABS 630 nm: Absorbance (read at 630 nm)

The corresponding immune responses are shown in Fig. 4. Each subfigure contains 4 vertical bars related to the strength of immune responses, and higher bars imply higher antigenicity and higher immune responses induced by Rabbit anti-GIVmcp and Rabbit anti-TGIVmcp antibodies. The first and the third bars in each subfigure are for the pre-immune serum before rGIVmcp and rTGIVmcp immunization, while the second and the forth bars represent the immune responses induced by Rabbit anti-GIVmcp antibody and Rabbit anti-TGIVmcp antibody. After observing the results of antigenicity test by ELISA assays, the exclusive LE segments obtained from the VIV subfamily (*Lymphocycstivirus*_157–166_, *Megalocycstivirus*_157–166_, and *Ranavirus*_152–161_) indeed induced significant immune responses (Fig. 4 a, b and c), while the exclusive LE segments obtained from the IIV subfamily of VALPTAALPYNE for *Iridovirus*_193~203_, VSLPTAALPYNE for *Chloriridovirus*_92~102_, VASQTVVPVVG for *Iridovirus*_226~236_, and TTGNPYQTIDV for *Chloriridovirus*_125–135_ reflect little cross host group reaction after immunization (Fig. 4 d, e, f, and g). In other words, both predicted exclusive LEs from IIV subfamily evoked little response to Rabbit anti-GIVmcp antibody and Rabbit anti-TGIVmcp antibody after ELISA assay validation. These results demonstrate important evidences of host-specific features for facilitating LE prediction and for effective vaccine development.

## Discussion

A voting mechanism-based LE prediction system for host-specific antigens is proposed. For group feature detection, the antigen sequences could be clustered prior to importing the sequences into the proposed system. Taking the *Iridoviridae* family as an example, the family can be categorized into two different subfamilies, vertebrate iridovirus and invertebrate iridovirus subfamilies. In this study, we applied different combinations of clustered groups to predict the conserved and exclusive LEs. The imported antigen sequences of each subfamily were analyzed by a consensus voting mechanism respectively. Protein structure prediction for query antigens was performed to conjecture whether the predicted LEs are located on protein structural surface, and a multiple structure alignment analysis was also performed to reconfirm the conserved and exclusive characteristics. Using the multiple sequence aligned locations, the consensus voting module selected epitope candidate residues by accumulating votes provided by five different renowned epitope prediction tools. In addition to individually voted epitope residues, the minimum lengths of concatenated epitope residues were also required for further experimental design. All LE candidates for different host specific groups were selected and cross-identified. In addition, to increase the successful results of vaccine design, we emphasized the surface structure characteristics of the predicted LE epitopes. Therefore, the antigens without resolved structures will be analyzed by Phyre^2^ for creating corresponding virtual structures. The predicted epitopes would be checked for their surface conditions based on the predicted structures, and the predicted structures will be structurally aligned by any structure alignment system for reconfirming exclusiveness of predicted LEs. Based on the aligned results, the predicted conserved and/or exclusive LEs for different subfamilies can be structurally distinguished. In order to validate the predicted LE segments, the two frequent iridovirus infections (TGIV/GIV) in Taiwanese groupers were especially applied for experimental validation. A total of five predicted LEs were synthesized and investigated for antigenicity and cross-antigenicity tests. The exclusive LE candidates for vertebrate subfamily including *Lymphcystivirus*_157–166_ (exclusive), *Megalocystivirus*_157–166_ (exclusive), and *Ranavirus*_152–161_ (exclusive) were selected as representative LEs for antigenicity tests against vertebrate hosts, while the LE candidates for invertebrate subfamily including *Chloriridovirus*_92–102_ (exclusive), *Chloriridovirus*_125–135_ (exclusive), *Iridovirus*_193–203_ (exclusive), and *Iridovirus*_226–236_ (exclusive) were selected as exclusive LEs for invertebrate group for cross antigenicity tests against vertebrate hosts. According to the ELISA results, it showed the exclusive LE segments for VIV subfamily indicated high antigenicity after immunity test against vertebrate hosts and the exclusive LE segments for invertebrate *Iridoviridae* segments showed no or little antigenic responses. All these experimental results imply the predicted LEs for VIV subfamily possessing relatively high antigenicity responses either for conserved (data not shown) or exclusive peptides, since both antigens (GIVmcp or TGIVmcp) affect the same host of groupers. We further compared the predicted LE peptides in the Table 2 and Table 3, and found that all conserved LEs predicted in two-clustered groups (VIV and IIV) are the same as the predicted LEs by taking the whole *Iridoviridae* family as a single group. Though these predicted conserved LEs indeed possessed high antigenicity and confirmed by five well known LE predictors, they would not practically evoke the immune responses or cause clinical signs of disease in vertebrate hosts. Hence, these conserved LEs obtained from *in-silico* prediction may be ignored for the following vaccine development stage, though these peptides also possess relatively high antigenicity. Furthermore, we investigated immune responses of the predicted exclusive LEs from IIV against vertebrate hosts, and the results showed there was no significant response. This is mainly due to both *Megalocystivirus* and *Ranavirus* belong to vertebrate host specificity, and this proved our assumption of host specific characteristics. Regarding biological validation experiments, commercial ELISAs might not be able to reflect pertinent responses for the predicted and synthesized LE peptides. To deal with such issues, a lot of iridovirus vaccines were evaluated through inactivated viruses [26–29], virus like particles (VLPs) [30], recombinant coat proteins [31–33] and DNA vaccines [34, 35]. However, there still exist several limitations to overcome such as structures of synthesized protein segments and the length settings of final predicted segments. These factors might affect the immune specificity, and different virus species might result in different protective efficacy. Therefore, this study could provide a precision approach for peptide vaccine design in iridovirus disease control, which can accelerate the process of vaccine development, reduce costs and save time. In some previous studies, adding linkers improved bioreactivity, folding, and stability of fusion proteins, such as helical peptide linkers, [A (EAAAK)nA] m (*n* = 2–4, m = 1 or 2) [36], or a flexible linker, (GGGGS)_3_ [37]. Some linkers can be used as adjuvants to enhance immune specificity for peptide vaccines [38–41]. Therefore, addition of suitable linkers to the predicted peptides could enhance and promote immune responses in hosts. We will further apply adjuvant-based linkers to predicted LE segments and enhance the specificity for immune responses in the near future. According to the results of the biological experiments and bioinformatics analysis, the developed voting mechanism-based linear epitope prediction system can successfully predict linear epitopes with significant antigenic specificity.

## Conclusions

We have developed voting mechanism-based linear epitope prediction for the host-specific *Iridoviridae* family. We used five renowned LE prediction systems and exclusive features, endorsed LE candidates with a minimum length requirement could be identified for each subfamily by various prediction systems. Our result suggests that there are some exclusive antigen features between the sequence of invertebrate Iridovirus and vertebrate Iridovirus which cause the host-specific features between these two groups. Furthermore, we apply surface comparison and ELISA to identify vertebrate host-specific LEs for both TGIV and GIV which reflect high antigenicity response for specific grouper species. According to this novel prediction system, the predicted LEs can facilitate immunologists in designing specific biological experiments for future vaccine development.

## Abbreviations

AIR: Alignment Incorporate Refinement
CE: Conformational epitope
GIV: Grouper Iridovirus
ICTV: International Committee on Taxonomy of Viruses
IIV: Invertebrate iridovirus
LE: Linear epitope
MCP: Major capsid protein
RMSD: residue-based root-mean-square-deviation
TGIV: Taiwan grouper Iridovirus
VIV: Vertebrate iridovirus
